# Altered Expression of Insulin Receptor Isoforms in Breast Cancer

**DOI:** 10.1371/journal.pone.0026177

**Published:** 2011-10-26

**Authors:** Jiaqi Huang, Chris Morehouse, Katie Streicher, Brandon W. Higgs, Jin Gao, Meggan Czapiga, Anmarie Boutrin, Wei Zhu, Philip Brohawn, Yong Chang, Jaye Viner, Theresa LaVallee, Laura Richman, Bahija Jallal, Yihong Yao

**Affiliations:** MedImmune, LLC., Gaithersburg, Maryland, United States of America; University of Nebraska Medical Center, United States of America

## Abstract

**Purpose:**

Insulin-like growth factor (IGF) signaling through human insulin receptor isoform A (IR-A) contributes to tumorigenesis and intrinsic resistance to anti-IGF1R therapy. In the present study, we (a) developed quantitative TaqMan real time-PCR-based assays (qRT-PCR) to measure human insulin receptor isoforms with high specificity, (b) evaluated isoform expression levels in molecularly-defined breast cancer subtypes, and (c) identified the IR-A:IR-B mRNA ratio as a potential biomarker guiding patient stratification for anti-IGF therapies.

**Experimental Design:**

mRNA expression levels of IR-A and IR-B were measured in 42 primary breast cancers and 19 matched adjacent normal tissues with TaqMan qRT-PCR assays. The results were further confirmed in 165 breast cancers. The tumor samples were profiled using whole genome microarrays and subsequently subtyped using the PAM50 breast cancer gene signature. The relationship between the IR-A:IR-B ratio and cancer subtype, as well as markers of proliferation were characterized.

**Results:**

The mRNA expression levels of IR-A in the breast tumors were similar to those observed in the adjacent normal tissues, while the mRNA levels of IR-B were significantly decreased in tumors. The IR-A:IR-B ratio was significantly higher in luminal B breast cancer than in luminal A. Strong concordance between the IR-A:IR-B ratio and the composite Oncotype DX proliferation score was observed for stratifying the latter two breast cancer subtypes.

**Conclusions:**

The reduction in IR-B expression is the key to the altered IR-A:IR-B ratio observed in breast cancer. The IR-A:IR-B ratio may have biomarker utility in guiding a patient stratification strategy for an anti-IGF therapeutic.

## Introduction

The mature human insulin receptor (IR) has two isoforms, insulin receptor isoform A (IR-A) and insulin receptor isoform B (IR-B). Both isoforms result from alternative splicing of the same primary transcript. IR-A differs from IR-B by the exclusion of exon 11, which encodes a 12 amino acid fragment (residues 717–728) of the insulin receptor α-subunit [1]. The relative abundance of mRNAs encoding the IR-A and IR-B isoforms is regulated in a tissue-specific manner [2] and also differs by stage of cell development and differentiation. IR-A is the predominant isoform in fetal tissues and cancer cells [3]. The two IR isoforms have been reported to exhibit distinct functional properties. While IR-B homodimers are the classical receptors for insulin with metabolic effects in muscle, liver and adipose tissues, IR-A homodimers can bind IGF-II and, to a lesser extent, IGF-I, in addition to insulin [4], [5], [6] and may be involved in mediating the growth promoting and anti-apoptotic effects of this growth factor under physiological conditions like embryonic development [3].

The fine regulation of the IR-A:IR-B expression ratio may be rendered more complex by coexpression of the cognate IGF1R, which may form hybrid receptors with IRs. Several studies have demonstrated the existence of IR-A/IGF1R hybrid receptors. These hybrid receptors are heterodimers comprised of an αβ chain from the IR and an αβ chain from the IGF1R, both in normal [7], [8], [9] and cancer cells [10]. Increased occurrence of these hybrids can be observed in several tumor cells as the result of IR and/or IGF1R overexpression [10], [11]. The pharmacological properties of these hybrids seem to differ from those of their homodimer counterparts and depend on the IR isoform involved. The IR-A/IGF1R hybrid receptors are strongly activated by IGF-I and IGF-II and weakly activated by insulin. By contrast, hybrid receptors containing IGF1R and IR-B are much less sensitive to IGF-II and insulin [12]. It is suggested that proliferative IGF signaling can occur through IGF1R homodimers, IGF1R/IR-A heterodimers, and IR-A homodimers [6], [13], [14].

As a key signaling component of the IGF pathway, IGF1R is the target of several investigational agents in clinical development. These agents were developed to specifically inhibit IGF1R while sparing IR-A due to concerns that co-targeting IGF1R and IR would result in unacceptable toxicity [15]. However, clinical failures with IGF1R-specific therapy suggest that it may be critical to inhibit aberrant IR-A signaling in addition to IGF1R. This assertion has been supported by Ulanet, et al. [15], who showed that IGF-II signaling through IR-A contributes to tumorigenesis and intrinsic resistance to anti-IGF1R therapy. Additional work supporting the importance of IR-A signaling was reported recently by Gao et al. [16], who described preclinical data using a monoclonal antibody that sequesters IGF1 and IGF2, thereby blocking signaling through both IGF1R and IR-A. Results showed that in response to blocking IGF1 and IGF2, dramatic antitumor activities were observed in tumors that express both IGF1R and IR-A or IR-A alone. Similar antitumor activities were not seen in these tumors when inhibiting IGF1R alone.

As therapeutic strategies for co-targeting IGF1R and IR-A move forward in clinical development, evaluating IR-A levels in clinical tumor samples becomes critical for identifying the most appropriate population to receive IGF-targeted therapy. To date, the most common method to specifically measure human IR-A and IR-B expression has been described by Frasca and colleagues [3]. This method is based on PCR amplification and gel separation, followed by qualitative measurement of the resulting bands. The method is tedious and lacks quantitative accuracy, which limits its potential use as a biomarker for clinical development. Due to the lack of an efficient and accurate method to detect human IR-A and IR-B mRNA levels, the expression of IR-A and IR-B in cancer tissues, particularly the IR-A:IR-B ratio, has been difficult to evaluate. In this paper, we describe the design and implementation of TaqMan qRT-PCR-based assays to specifically quantify the levels of IR-A and IR-B transcripts in human breast cancer samples. Furthermore, we evaluated the ratio of IR-A to IR-B expression in ER+/PR+ and Her2- breast cancers of different molecular subtypes. As these subtypes have previously been shown to differ in proliferation index, response to hormonal therapy, and overall clinical outcome, the association of the IR-A:IR-B ratio with a particular subtype may identify patients more likely to respond to IGF-targeted therapy.

## Materials and Methods

### Primary Breast Tumor Tissues

Forty-two grade I to III infiltrating breast ductal carcinomas were purchased from ILSbio (Chestertown, MD). Nineteen of the 42 breast tumor tissue samples had matched normal adjacent breast tissue (NAT) samples. In the case of breast cancer mastectomy, normal tissue from the opposite breast was taken and serves as the normal adjacent tissue. The ages of patients ranged from 31 to 88 years. All the breast cancer samples were estrogen receptor (ER) and progesterone receptor (PR) positive and HER2 negative according to immunohistochemistry. All samples were freshly frozen and collected before initiation of any treatment. Tumor samples were macrodissected to remove normal tissue, and tumor purity in all samples was greater than 85%. Normal samples were macrodissected to remove non-glandular tissue.

### Breast Cancer Tissue cDNA Arrays

Four breast cancer tissue cDNA arrays (BCRT101, BCRT102, BCRT103, and BCRT104) were purchased from OriGene Technologies (Rockville, MD). The arrays contain cDNAs from 15 normal breast tissues (from 10 unique individuals) and 165 unique breast adenocarcinoma tissues. The tumor stage ranged from stage I to IV and the tissues were comprised of 50–90% tumor.

### RNA Extraction

Total RNA was extracted from snap-frozen tissue specimens using the ZR RNA MicroPrep kit (Zymo Research, Orange, CA). RNA purity and concentration were determined spectrophotometrically (260/280>1.9). RNA quality was assessed on an Agilent 2100 Bioanalyzer using the RNA 6000 Nano LabChip®.

### Design Real-time Quantitative PCR Primer and Probes for Insulin Receptor Isoforms

Full-length mRNA transcript sequences for the IR-A (NM_001079817) and IR-B (NM_000208) isoforms were retrieved from the NCBI Reference Sequences database. For the IR-A assay design, we targeted the exon 10/12 junction region for the gene specific probe; the exon 10 coding region for the forward primer pairs; and the exon 12 coding regions for the reverse primer pairs. For the IR-B assay design, we targeted the exon 11 interior coding region for the gene specific probe; exon 11/12 junction for the forward primer pairs; and exon 12 for the reverse primer pairs. All primer/probe designs were imported into the Primer Express (Applied Biosystems, Foster City, CA) software tool to ensure optimal design for utilization in the TaqMan Gene Expression assay procedure. All probes were designed to incorporate a minor groove binding moiety (MGB), and were labeled with a fluorescent dye (FAM) for detection and a non-fluorescent quencher. Primers and probes were custom ordered from Applied Biosystems. Sequences for all primer/probe combinations are as follows:

IR-A: The sequence of the probe is 5′-TCCCCAGGCCATCT -3′;

The sequence of the forward primer is 5′-TGAGGATTACCTGCACAACG -3′;

The sequence of the reverse primer is 5′- ACCGTCACATTCCCAACATC -3′.

IR-B: The sequence of the probe is 5′ CCGAGGACCCTAGGC -3′;

The sequence of the forward primer is 5′- CGTCCCCAGAAAAACCTCTTC -3′;

The sequence of reverse primer is 5′-GGACCTGCGTTTCCGAGAT -3′.

### Positive and Negative Controls for TaqMan assay of IR-A and IR-B

Commercially available cDNA clones that contain the full-length cDNA clones of IR-A (cloned in pCMV6-XL4) and IR-B (cloned in pCMV6-XL5) were purchased from OriGene Technologies, Inc (IR-A: SKU#. SC311328; IR-B: SKU# SC315880). The sequence verification of each IR isoform clone was conducted in-house at MedImmune. The empty plasmids pCMV6-XL4 and pCMV6-XL5 were used as negative control DNA for IR-A and IR-B assays, respectively.

### TaqMan qRT-PCR Gene Expression Analysis

Standard TaqMan qRT-PCR Gene Expression assays were conducted in a 384-well format for all primer/probe and template combinations. Reactions consisted of 5 µL of TaqMan Universal Master Mix (Applied Biosystems), 0.5 µL of 10x Gene Expression Assay Mix, and 4.5 µL of varying copy numbers of either the IR-B or IR-A cDNA clone, for a final volume of 10 µL per well. Each primer/probe and template combination was repeated at least 3 times. All assay plates were run on an Applied Biosystems 7900HT detection system using standard settings (cycling program included 10 min incubation at 95°C followed by 40 cycles of 95°C for 15 sec and 60°C for 1 min). Data values (Cycle Threshold [Ct] values) were extracted from each assay with the SDS v2.0 software tool (Applied Biosystems).

### Assessment of the Expression levels of Other Genes in Breast Cancer

TaqMan Gene Expression assays were purchased from Applied Biosystems. The assays include: INSR (Assay ID: Hs00961554_m1); ESR1 (Assay ID: Hs00174860_m1); PGR (Assay ID: Hs01556707_m1); ERBB2 (HER2, Assay ID: Hs01001580_m1); tumor proliferation genes (Pike S et al 2004): BIRC5 (Survivin, Assay ID:Hs00153353_m1), AURKA (STK15, Assay ID:Hs01582073_m1), CCNB1 (Assay ID: Hs00259126_m1), MKI67 (Assay ID: Hs01032443_m1), MYBL2 (Assay ID: Hs00942543_m1); and reference genes: ACTB (Hs99999903_m1), GUSB (AssyID:Hs99999908_m1), GAPDH (Assay ID: Hs99999905_m1), RPLP0 (Assay ID: Hs99999902_m1), TFRC (Assay ID: Hs99999911_m1).

### BioMark™ Dynamic Array Microfluidics System

The BioMark™ Dynamic Array (Fluidigm Corporation) microfluidics system allows for high throughput real-time PCR (up to 2304 individual reactions per plate), producing high quality data with low variability and a tight correlation with conventional RT-PCR. Single stranded cDNA was generated from total RNA using the SuperScript® III First-Strand Synthesis SuperMix (Invitrogen, Carlsbad, CA). cDNA samples were pre-amplified using TaqMan Pre-Amp Master Mix, according to the manufacturer's instructions. Reactions contained 5 µL of cDNA, 10 µL Pre-Amp Master Mix and 5 µL of 0.2X gene expression assay mix (comprised of all primer/probes to be assayed) for a final volume of 20 µL. Reactions were cycled with the recommended program for 14 cycles and then diluted 1∶5 with TE buffer. Pre-amplified cDNA was either utilized immediately or stored at −20°C until processed.

To prepare samples for loading into 48×48 dynamic array chips (Fluidigm), the reaction mix contained 2.5 µL 2X Universal Master Mix (Applied Biosystems), 0.25 µL Sample Loading Buffer (Fluidigm Corporation), and 2.25 µL pre-amplified cDNA. To prepare the primer/probes, the reaction mix contained 2.5 µL 20X TaqMan Gene Expression Assay and 2.5 µL Assay Loading Buffer (Fluidigm Corporation). Prior to loading the samples and assay reagents into the inlets, the chip was primed in the IFC Controller. Five µL of sample prepared as described was loaded into each sample inlet of the dynamic array chip, and 5 µL of 10X gene expression assay mix was loaded into each detector inlet. The chip was placed on the IFC Controller for loading and mixing. Upon completion of the IFC priming step, the chip was loaded on the BioMark™ Real-Time PCR System for thermal cycling (10 min at 95°C followed by 40 cycles of 95°C for 15 sec and 1 min at 60°C). The number of replicates and the composition of the samples varied depending on the particular experiment, but were never less than triplicate. Average Ct values were used to determine sensitivity and specificity of the designed probes. The average Ct values of all available reference gene assays within a sample were utilized for ΔCt calculation.

### Microarray Processing

Generation of biotin-labeled amplified cRNA from 75 ng of total RNA was accomplished with the MessageAmp^TM^ Premier RNA Amplification Kit (Ambion, Austin, TX). The concentration and purity of the cRNA product were determined spectrophotometrically. Fifteen micrograms of each biotin-labeled cRNA was fragmented for hybridization on Affymetrix Human Genome U133 Plus 2.0 GeneChip® arrays. All GeneChip® washing, staining, and scanning procedures were performed with Affymetrix standard equipment. Data capture and initial array quality assessments were performed with the GeneChip Operating Software (GCOS) tool. Stratagene's (La Jolla, CA) ArrayAssist® Lite software was used to calculate probe-level summaries (GC-RMA and MAS5) from the array CEL files.

### Breast Cancer Molecular Subtype Classification

A subset of ER+/PR+ and HER2- primary breast tumors (n = 40) and a subset of matched normal adjacent breast tissue samples (n = 15) were profiled on Affymetrix Human Genome U133 Plus 2.0 GeneChip® arrays. Two of the primary breast samples and four of the normal adjacent breast tissue samples analyzed on the Fluidigm platform were not processed on GeneChip due to insufficient RNA quantity. Breast cancer molecular subtype classification, with regards to luminal-A and luminal-B subtype, was conducted utilizing our whole genome array data.

Two methods for determining putative sample classification were implemented. The first classification method utilized a published PAM50-gene shrunken centroid classifier [17] for sample sub-typing (normal, basal-like, HER2, luminal-A, or luminal-B) purposes. MAS5 normalized GeneChip data was used for this analysis given that the published classifier was built using this type of scaled data. The samples were classified according to a Spearman's rank correlation (50-gene intensity vector vs. subtype centroid classifier), where the subtype with the highest correlation value was assigned to a particular sample. The second method utilized GC-RMA normalized GeneChip data to identify a panel of differentially expressed transcripts by a two-sample Welch's t-test analysis. Samples were divided into two groups (normal or tumor) based on pathology assessment prior to conducting the statistical analysis. Probes displaying a fold change differential >3 and *P*-value <1.0×10^−12^ (*q*-value <1.0×10^−11^) (n = 459 probes) were used for an unsupervised hierarchical clustering analysis. Several additional probeset selection thresholds were utilized and resulted in similar clustering patterns (data not shown).

## Results

### Sensitivity and Specificity of TaqMan qRT-PCR Assay to Distinguish IR-A and IR-B

**Figure 1:**
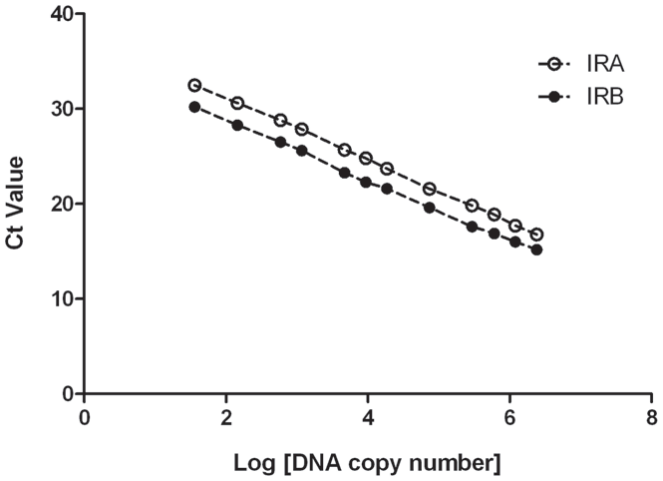
IR-A and IR-B assay serial dilution curves. Probe sensitivity was tested by performing IR-A or IR-B TaqMan qRT-PCR assays with a serial dilution of approximately 10^7^ to 10 copies of plasmid DNA of either IR-A, IR-B, or an empty vector control. The Y axis represents cycle-threshold (Ct) values and the X axis represents log DNA copy number. The slope and regression coefficient (*r* ^2^ value) of the standard dilution curve for the IR-A assay are -3.259 and 0.9992, respectively. The slope and regression coefficient (*r* ^2^ value) of the standard dilution curve for the IR-B assay are -3.155 and 0.9989, respectively.

Probe sensitivity was tested by performing IR-A or IR-B TaqMan qRT-PCR assays starting with 100 pg template stock solutions of IR-A or IR-B (approximately 10^7^ copies of DNA template). The DNA was serially diluted to 10^−4^ pg (approximately 10 copies DNA template). Each sample was tested in duplicate. The slope of the standard template dilution curve was determined by plotting cycle-threshold (Ct) values as a function of the log DNA copy number. The results are shown in Figure 1. Strong correlations were observed between the log [concentration] and resultant Ct values for each assay tested with its respective matching standard template. All regression coefficients (*r* ^2^ value) were ≥0.999 (*P*≤0.0001). Linearities were maintained in the DNA concentration ranges described above in both assays, demonstrating a wide dynamic range and yielding accurate Ct values. The results indicate that both the IR-A and IR-B assays have the appropriate sensitivity, and are able to detect the corresponding isoform to approximately 35 copies of DNA.

The specificity of the assays was also assessed by testing the IR-A assay in the presence of the IR-B DNA template or, alternatively, the IR-B assay in presence of the IR-A DNA template. The details of the testing results are shown in Table S1. The IR-A assay does not amplify IR-B DNA template in the range tested (10 to 10^7^ copies of IR-B DNA). Likewise, the IR-B assay does not amplify the IR-A DNA template in the range tested. In contrast, we found the commercial Applied Biosystems assays (IR-A: Hs00965956_m1; IR-B: Hs00169631_m1) had strong cross-interaction and could not measure each isoform accurately (data not shown).

The assay efficiency was assessed by the slopes of the standard dilution curves for both assays (Figure 1). The slope is -3.259 for IR-A and -3.155 for IR-B. The two slopes are very similar, suggesting only minor differences in probe efficiency.

These TaqMan-based assays have been successfully implemented on the BioMark™ Dynamic Array (Fluidigm), facilitating their use in a higher throughput system and demonstrating their performance across multiple platforms.

### Expression of IR-A and IR-B in Breast Cancer

#### Decreasing IR-B mRNA expression level in tumors

**Figure 2:**
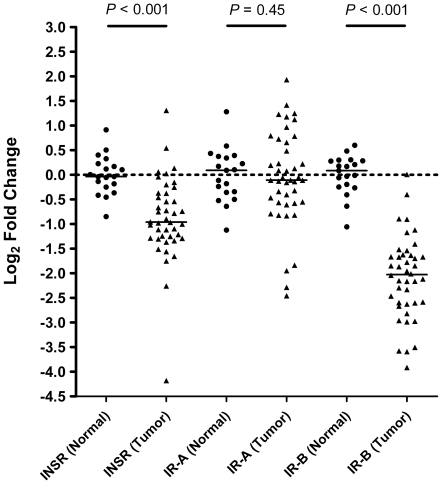
Relative mRNA expression levels of insulin receptor and its isoforms in primary breast cancer compared to normal breast tissues. TaqMan gene expression assay determined fold change differentials (log_2_-base scale) of INSR (total), IR-A, and IR-B between normal (n = 19) and tumor (n = 42) breast tissue samples. Average normal ΔCt values were used for calculation of fold change differentials for each sample. A two-tailed Welch's t-test analysis identified a significant difference between normal and tumor samples for both INSR and IR-B (*P*<0.001), whereas no difference was observed for IR-A (*P* = 0.450). Black bars represent the median log_2_ fold change value within a particular gene target and tissue-type combination.

In order to assess the IR-A and IR-B mRNA expression in breast cancer, 42 primary breast tissue samples that are ER+, PR+ and HER2-, and 19 matched normal adjacent breast tissues were evaluated. Random hexamer primed cDNAs were pre-amplified and assayed for expression levels of total IR, IR-A, and IR-B transcripts by TaqMan qRT-PCR (Fluidigm). Samples were normalized to the average of five housekeeping genes as described in the methods section. The results are shown in Figure 2. The mean fold change differentials (log2-base scale) ±95% CI of IR, IR-A, and IR-B in normal (n = 19) were 0.00±0.20, 0.00±0.27, and 0.00±0.20, respectively. The mean fold change differentials (log2-base scale) ±95% CI of IR, IR-A, and IR-B in tumor (n = 42) were -0.88±0.29, -0.07±0.33, and -2.08±0.29, respectively. A two-tailed Welch's t-test analysis indicated that the mRNA levels of IR and IR-B are significantly lower in breast cancer when compared to normal breast tissue (*P<*0.0001). No significant differences were observed in the mRNA levels of IR-A in breast cancer when compared with normal breast tissue (*P* = 0.450).

#### Increasing the relative proportion of IR-A in tumors

**Figure 3:**
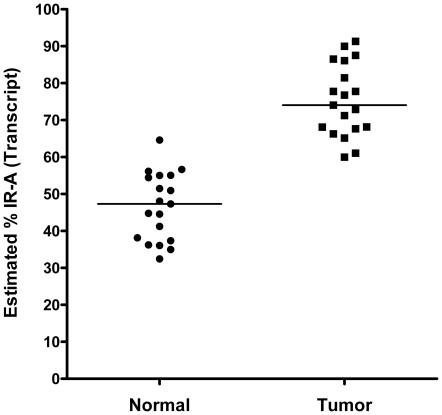
The proportion of IR-A expression in matched normal breast and breast cancer specimens. Within sample proportion of insulin receptor isoform-A (IR-A) relative to total insulin receptor composition (ie, IR-A + IR-B) as determined by 2^(-ΔCt)^ calculation. A paired sample t-test analysis indicated that a significant difference exists for calculated IR-A proportion between matched normal and tumor samples (*P*<0.001). Black bars represent the median IR-A proportion (%) within the normal (46.60±4.74, mean %±95% CI) and tumor (75.24±5.03, mean %±95% CI) tissue.

We calculated the proportion of IR-A relative to total insulin receptor composition (IR-A + IR-B) in matched tumor and normal pairs as determined by a 2^(−ΔCt)^ calculation. The results are shown in Figure 3. The mean IR-A transcript proportions (%)± 95% CI were 46.60%±4.74% and 75.24%±5.02% for the normal panel (n = 19) and matched tumor samples, respectively. A paired sample t-test analysis indicated that a significant increase in the calculated IR-A proportion in tumor samples exists when compared to matched normal tissue (*P*<0.0001). The results suggest that the significantly decreased IR-B levels in tumor contribute to an overall increase in the proportion of IR-A in tumor samples compared to normal tissue.

#### Ratios of IR-A to IR-B in breast cancer

**Figure 4:**
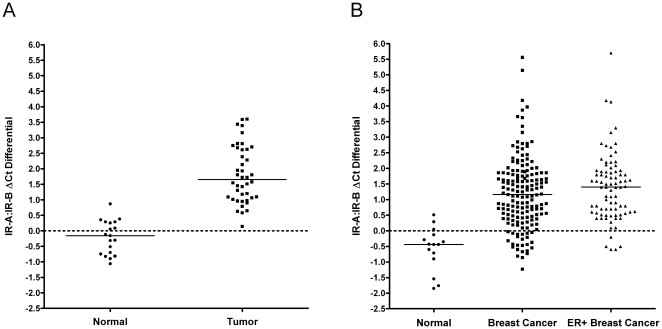
Increasing IR-A:IR-B ratio in primary breast cancer and breast cancer samples from qPCR cDNA array. (A) Calculated ΔCt differentials of insulin receptor isoforms IR-A and IR-B in primary breast cancer samples: normal (n = 19) and tumor (n = 42) breast samples. (B) Calculated ΔCt differentials of insulin receptor isoforms IR-A and IR-B breast cancer samples from qPCR cDNA array: normal (n = 15), breast cancer cDNA panels (n = 168), and breast tumors from these cDNA panels with >2-fold estrogen receptor over-expression (n = 83). ΔCt differentials (IR-B ΔCt - IR-A ΔCt) values were calculated for all samples utilizing the within-sample reference gene panel (average Ct) for normalization purposes. A two-tailed Welch's t-test analysis identified a significant difference between normal and tumor samples in relation to observed IR-A:IR-B ΔCt differential (*P*<0.001). Black bars represent the median IR-A:IR-B ΔCt.

In order to assess the mRNA transcript ratios of IR-A and IR-B, we calculated ΔCt differentials of IR-A and IR-B in normal and primary breast tumor samples. The ΔCt differential (IR-B ΔCt–IR-A ΔCt) values were calculated for all samples utilizing the within-sample reference gene panel (average Ct) for normalization purposes. The mean IR-A:IR-B ΔCt ±95% CI were -0.20±0.27 and 1.81±0.31 in the normal (n = 19) and primary tumors (n = 42), respectively. A two-tailed Welch's t-test analysis identified a significant difference between normal and tumor samples in relation to observed IR-A:IR-B ΔCt (*P*<0.0001) (Figure 4A). The results indicate that there is a significant increase in the ratio of IR-A to IR-B in breast tumors.

**Figure 5:**
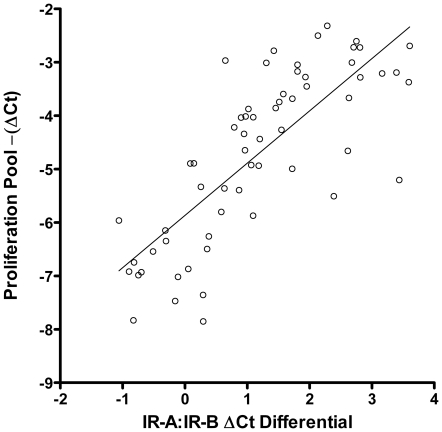
Correlation of IR-A:IR-B ΔCt differential with the expression of proliferation genes. Linear regression analysis of the relationship between calculated IR-A:IR-B ΔCt differential (X axis) and a pooled panel of proliferation markers (AURKA, BIRC5, CCNB1, KI67, and MYBL2) (Y axis). Proliferation panel summary values were calculated by taking the mean –(ΔCt) across all markers for a particular sample. Summary results for both normal and tumor samples are presented. The linear regression analysis results suggest a positive correlation between the two summary values (ρ = 0.78).

To further validate the above results, we assessed mRNA expression ratios of IR-A and IR-B in an additional breast cancer tissue panel. PCR arrays containing cDNAs from 15 normal breast tissues (obtained from 10 independent donors) and 165 unique breast adenocarcinoma tissues were used. Equal amounts of cDNA were pre-amplified and assayed for expression levels of IR-A, IR-B, and ER by TaqMan qRT-PCR (Fluidigm). The ΔCt differential (IR-B ΔCt–IR-A ΔCt) was calculated for all samples utilizing the within-sample reference gene panel (ACTB, GUSB, and GAPDH) for normalization purposes. The results are shown in Figure 4B. The mean IR-A:IR-B ΔCt ±95% CI was -0.57±0.34 in normal tissues (n = 15); and 1.20±0.17 across all breast cancers examined (n = 165). We then collected the breast cancer samples that displayed a 2-fold overexpression of estrogen receptor relative to normal breast tissue, and compared their IR-A:IR-B ΔCt differentials to normal tissue and to all breast cancer samples. The results are shown in Figure 5. The mean IR-A:IR-B ΔCt ±95% CI was 1.39±0.23 in ER+ breast cancers (n = 83), which is similar to that observed across the whole breast cancer dataset. A two-tailed Welch's t-test analysis identified a significant difference between normal and tumor samples in relation to observed IR-A:IR-B ΔCt differential (*P*<0.0001).

### Correlating IR-A:IR-B Ratio with Genes Involved in Breast Cancer Proliferation

AURKA, BIRC5, CCNB1, MKI67 and MYBL2 are well characterized genes involved in breast cancer proliferation [18]. The composite expression score of these genes has been used in Oncotype DX and cell proliferation is the most important factor contributing to breast cancer recurrence in many patients. Therefore, we investigated the relationship of the IR-A:IR-B ratio with the proliferation score in primary breast cancer sample sets using regression and correlation analyses. Linear regression analysis was conducted to quantify the relationship between the calculated IR-A:IR-B ΔCt differential and a pooled panel of proliferation markers (AURKA, BIRC5 , CCNB1, MKI67, and MYBL2). Proliferation panel summary values were calculated by taking the average ΔCt across all markers for a particular sample. Summary results for both normal and tumor samples are presented in Figure 5. The linear regression analysis results suggest a moderate positive correlation between the two summary values (ρ = 0.78). These results suggest that the increased proportion of IR-A observed in tumor tissue may contribute to enhanced proliferation in ER+/PR+ and HER2- breast cancer.

### IR-A:IR-B ΔCt Differential in Breast Cancer Subtypes

Using mRNA expression profiles, ER+ breast cancers can be further classified by hierarchical cluster analysis into luminal-A and luminal-B subtypes [19]. Luminal-A cancers are usually histologically low-grade and sensitive to endocrine therapy [20]. By contrast, luminal-B cancers are often high-grade and are less sensitive to endocrine therapy, and have higher proliferation and poorer prognosis [20], [21]. Creighton, et al. [22] reported that an IGF-I signature is manifested in luminal-B breast cancers. This signature is highly correlated with poor prognostic factors and one of the strongest indicators of disease outcome. To examine the role of additional components of the IGF signaling pathway in breast cancer, we investigated the hypothesis that differences in IR-A:IR-B ratios may be evident when comparing luminal-A and luminal-B breast cancers.

**Figure 6:**
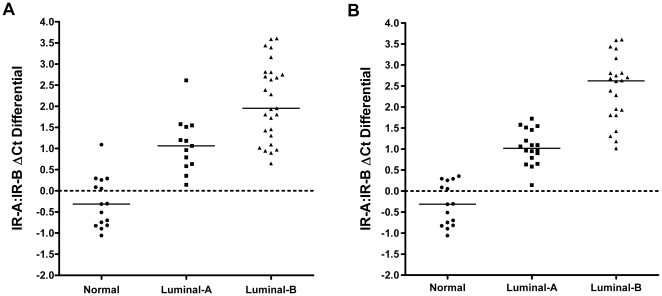
IR-A:IR-B ΔCt differential in subtypes of ER+ breast cancer. Scatter plot representation of calculated IR-A:IR-B ΔCt differentials with regard to sample subtype (normal, luminal-A, or luminal-B) classification determined by a shrunken centroid classifier-based methodology (A) and a hierarchical clustering analysis (B). All subtype pair-wise comparisons display a significant difference (two-sample t-test, *P*<0.001). Black bars represent the median IR-A:IR-B ΔCt differential within a particular sample subtype.

To address this question we conducted whole genome array analysis on 40 ER+/PR+ and HER2- negative breast tumors and 15 normal breast samples. We initially utilized a published PAM50-gene shrunken centroid classifier [17] on our MAS5 normalized GeneChip data as a benchmark for a method that has been used to partition ER+/PR+ and HER2- negative breast tumor samples into luminal-A and luminal-B subtypes. Samples were classified according to a Spearman's rank correlation, where the subtype with the highest correlation value was assigned to a particular sample. IR-A:IR-B ΔCt differentials in the normal, luminal-A and luminal-B samples were then compared. The scatter plot representation of calculated IR-A:IR-B ΔCt differentials with regards to sample subtype (normal, luminal-A, or luminal-B) are shown in Figure 6A. The mean IR-A:IR-B ΔCt ±95% CI was -0.27±0.34 in normal (n = 15); 1.09±0.38 in luminal-A breast cancers (n = 13); and 2.12±0.39 in luminal-B breast cancers (n = 27). All subtype pair-wise comparisons display a significant difference (two-sample t-test, *P*<0.001). The results indicate that IR-A:IR-B ratios are significantly increased in luminal-B cancers relative to luminal-A cancers, with the greatest differential observed between luminal-B and normal breast tissue. Additionally, we found that these luminal-B samples exhibited a significant increase in expression of a proliferation gene panel (data not shown). Increased proportion of IR-A in luminal-B patients may play an important role in inducing the increased proliferation observed in this study and others [21].

In addition to the shrunken centroid classifier, we identified an independent panel of differentially expressed transcripts from our GeneChip data using a two-sample Welch's t-test and unsupervised hierarchical clustering analysis. Samples were divided into two groups (normal or tumor) based on pathology assessments performed prior to conducting the statistical analysis. Sub-populations identified were classified as normal, luminal-A, or luminal-B as a function of transcript panel composition for the three primary clusters identified. Differentiation of luminal-A and luminal-B sub-types was primarily determined by over-expression of proliferation and cell cycle associated genes (e.g. AURKB, BUB1, CCNA2, CCNE2, E2F7, GTSE1, MKI67, and PKMYT1) in the putative luminal-B tumors and over-expression of extracellular matrix associated genes (e.g. ASPN, BGN, CILP2, MFAP2, and VCAN) in the putative luminal-A tumors. IR-A:IR-B ΔCt differentials in the normal, luminal-A and luminal-B were then compared. The results are shown in Figure 6B. The mean IR-A:IR-B ΔCt ±95% CI was -0.32±0.28 in normal (n = 15); 1.05±0.20 in luminal-A predicted breast cancers (n = 18); and 2.42±0.37 in luminal-B predicted breast cancers (n = 22). All subtype pair-wise comparisons display a significant difference (two-sample t-test, *P*<0.001).

Overall, an 80% concordance between the two classification methods implemented was observed, based on our unbiased assessments. Unfortunately, we are naïve to the true sub-type classification of the tumor samples so it is difficult to resolve differences in classification between the two methodologies. However, the overall concordance between the results from comparisons of the IR-A:IR-B ΔCt differential with both the published PAM50 classifier and an independent gene selection and clustering method further supports our observation that the IR-A:IR-B ratio is significantly increased in the luminal-B subtype of breast cancer.

## Discussion

We have described specific TaqMan qRT-PCR-based assays that accurately measure the mRNA levels of human IR isoforms IR-A and IR-B. In the absence of specific reagents to quantitatively distinguish the two isoforms, previous research utilized an RT-PCR/gel quantification protocol to distinguish IR-A and IR-B and measure their relative amounts in normal and cancer tissues [3]. The TaqMan assays we have developed provide a significant improvement in sensitivity, reproducibility, and quantitative capacity over previous methods. The assays have been successfully transferred to the BioMark™ Dynamic Array (Fluidigm) platform for high throughput applications, suggesting that migration to an applicable clinical testing platform is also possible. Additionally, preliminary results indicate that we can successfully implement these assays on FFPE samples due to the relatively small amplicon size of each assay (data not shown). Since FFPE is the clinical standard for cancer diagnosis, examining samples in this manner will allow for high-throughput and sensitive quantitation of IR-A:IR-B ratios over what is typically a long course of clinical care and also help identify the subset of patients with altered IR-A:IR-B ratios that might respond to IGF-targeted therapy.

Over-expression of hybrid IGF1R and IR-A receptors has been reported in breast cancer [23], but owing to technical difficulties in quantifying IR-A and IR-B in large numbers of clinical samples, the overall expression levels of IR-A and IR-B in breast cancer relative to normal breast tissue were not fully known. Our analysis of primary breast cancer samples indicated that IR-A mRNA expression levels in the breast tumors were similar to the levels observed in adjacent normal breast tissues, while the mRNA expression levels of IR-B were largely decreased when compared to the adjacent normal breast tissues. Our observation of an increased IR-A:IR-B ratio in breast cancer tissue relative to normal is consistent with what has been seen previously; however, our results are distinct in that we identified a reduction in IR-B expression as the key to this altered ratio. Given the link between altered expression of IR isoforms and resistance to the EGFR inhibitor gefitinib [24], it is clear that aberrant expression levels of IR-A and IR-B may have significant functional effects on cancer progression.

The functional consequences of an altered IR-A:IR-B ratio on cancer progression may also be mediated, in part, by changes in receptor dimerization patterns and the subsequent effect on cell signaling pathways. An increase in the relative abundance of IR-A might significantly increase formation of IGF1R and IR-A hybrid receptors, IR-A/IR-B heterodimers, and IR-A homodimers in breast cancer tissues. These changes in dimerization patterns could result in an increased number of binding sites for IGFs, leading to increases in mitogenic signaling in breast cancer cells [23], as well as changes in gene expression strongly associated with cell proliferation, metabolism, and DNA repair [22]. Of the genes altered in response to IGF stimulation, the mRNA expression levels of proliferation genes are most closely correlated with clinical outcomes [18], [25], [26]. Our data illustrate a positive correlation between the IR-A:IR-B ΔCt differential and the proliferation score. These data also provide supporting evidence that the increasing IR-A proportion may contribute to the tumor proliferation phenotype of breast cancers.

Gene expression profiling studies have consistently revealed biologically distinct breast cancer subtypes with different prognoses and treatment responses [27]. At the molecular level, ER+/PR+ and HER2- breast cancer can be further divided into luminal-A and luminal-B subtypes [19]. The luminal-B subtype is characterized by a cell proliferation signature that includes the expression of MKI67, CCNB1, and MYBL2, which have been associated with tamoxifen resistance [21], [28]. The luminal-A subtype, by contrast, is characterized by lower proliferation, response to hormonal therapy and relatively good prognosis. In this study we found a positive correlation between IR-A:IR-B ΔCt differential and the expression of multiple proliferation genes, as well as a more prominent IR-A:IR-B ΔCt differential in luminal-B breast cancers classified according to conventional methods. Therefore, our findings support the assertion that the significant increase of IR-A:IR-B ratio in luminal-B patients may be related to the high proliferation and poor prognosis observed for this subtype. The luminal-B subtype has been clinically associated with higher grade, larger tumor size, positive lymph node involvement, increased lymphovascular invasion, and poorer relapse-free survival [29]. Furthermore, breast cancer patients with luminal-B tumors are at increased risk for relapse and death, despite endocrine therapy [29]. The association of increased IR-A:IR-B ratio with the luminal-B subtype suggests that luminal-B patients could potentially respond to therapy that co-targets IGF1R and IR-A. Moreover, it highlights the potential importance of targeting IR-A in this subgroup of patients and suggests that therapeutic strategies designed to target the growth factors IGF1 and IGF2 themselves, may be more successful than strategies targeting the IGF1R only.

Previous work has shown that protein levels of the total insulin receptor are elevated in breast cancer tissue compared to normal breast tissue [30] and that insulin itself may affect tumor progression by acting through its own receptor [31]. Specifically, Papa et al. [30] reported the average protein expression level of the insulin receptor was elevated approximately six-fold in 159 breast cancer tissues when compared to 27 normal breast tissues. These results appear to contradict our results, which suggest that total insulin receptor transcript levels are lower in breast cancer tissue when compared to normal breast tissues. However, in a subsequent manuscript from the same group [31], total insulin receptor protein levels were shown to be elevated in 2 of 3 breast cancer cell lines, but no quantitative difference in insulin receptor mRNA content was observed in these same cell lines. This suggests that post-transcriptional mechanisms could play a role in regulating the total protein level of insulin receptor in cancer. In contrast, relative increases in the mRNA expression ratio of IR-A and IR-B has been observed in dedifferentiated thyroid carcinomas [32] and associated with characteristics of stemness and the development of thyroid cancer [33]. Therefore, alterations in the IR-A:IR-B transcript ratio observed in this study could significantly increase our understanding of the role of insulin receptor isoforms in breast cancer, independent of the total insulin receptor content in these tumors.

In summary, the specific TaqMan qRT-PCR-based assays that we developed can easily be incorporated into routine clinical practice and measure the relative abundance of IR-A and IR-B mRNAs in human tissues with high sensitivity and specificity. Studies based on these assays may provide value in evaluating the therapeutic benefit of targeting both IGF1R and IR-A pathways by inhibiting the growth factors IGF1 and IGF2, as well as potentially identifying patients more likely to respond to any anti-IGF therapy under clinical development. The results reported here provide a promising foundation to support these future efforts.

## Supporting Information

Table S1 **Specificity of IR-A and IR-B assays.** Assay specificity was tested by performing IR-A, IR-B, and IR qRT-PCR assays with a serial dilution of approximately 107 to 10 copies of plasmid DNA of IR-A and IR-B. Cross reactions of either IR-A assay to IR-B DNA or IR-B assay to IR-A DNA were not observed.
(PDF)

## References

[1] Seino S, Seino M, Nishi S, Bell GI (1989). Structure of the human insulin receptor gene and characterization of its promoter.. Proc Natl Acad Sci U S A.

[2] Moller DE, Yokota A, Caro JF, Flier JS (1989). Tissue-specific expression of two alternatively spliced insulin receptor mRNAs in man.. Mol Endocrinol.

[3] Frasca F, Pandini G, Scalia P, Sciacca L, Mineo R (1999). Insulin receptor isoform A, a newly recognized, high-affinity insulin-like growth factor II receptor in fetal and cancer cells.. Mol Cell Biol.

[4] Mosthaf L, Vogt B, Haring HU, Ullrich A (1991). Altered expression of insulin receptor types A and B in the skeletal muscle of non-insulin-dependent diabetes mellitus patients.. Proc Natl Acad Sci U S A.

[5] Sesti G, Marini MA, Tullio AN, Montemurro A, Borboni P (1991). Altered expression of the two naturally occurring human insulin receptor variants in isolated adipocytes of non-insulin-dependent diabetes mellitus patients.. Biochem Biophys Res Commun.

[6] Kellerer M, Sesti G, Seffer E, Obermaier-Kusser B, Pongratz DE (1993). Altered pattern of insulin receptor isotypes in skeletal muscle membranes of type 2 (non-insulin-dependent) diabetic subjects.. Diabetologia.

[7] Pandini G, Vigneri R, Costantino A, Frasca F, Ippolito A (1999). Insulin and insulin-like growth factor-I (IGF-I) receptor overexpression in breast cancers leads to insulin/IGF-I hybrid receptor overexpression: Evidence for a second mechanism of IGF-I signaling.. Clin Cancer Res.

[8] Vella V, Sciacca L, Pandini G, Mineo R, Squatrito S (2001). The IGF system in thyroid cancer: New concepts.. Mol Pathol.

[9] Pandini G, Medico E, Conte E, Sciacca L, Vigneri R (2003). Differential gene expression induced by insulin and insulin-like growth factor-II through the insulin receptor isoform A.. J Biol Chem.

[10] Haring HU, Kellerer M, Mosthaf L (1994). Modulation of insulin receptor signalling: Significance of altered receptor isoform patterns and mechanism of hyperglycaemia-induced receptor modulation.. Diabetologia.

[11] Soos MA, Siddle K (1989). Immunological relationships between receptors for insulin and insulin-like growth factor I. Evidence for structural heterogeneity of insulin-like growth factor I receptors involving hybrids with insulin receptors.. Biochem J.

[12] Pollak M (2008). Insulin and insulin-like growth factor signalling in neoplasia.. Nat Rev Cancer.

[13] Soos MA, Nave BT, Siddle K (1993). Immunological studies of type I IGF receptors and insulin receptors: Characterization of hybrid and atypical receptor subtypes.. Adv Exp Med Biol.

[14] Soos MA, Whittaker J, Lammers R, Ullrich A, Siddle K (1990). Receptors for insulin and insulin-like growth factor-I can form hybrid dimers. characterization of hybrid receptors in transfected cells.. Biochem J.

[15] Ulanet DB, Ludwig DL, Kahn CR, Hanahan D (2010). Insulin receptor functionally enhances multistage tumor progression and conveys intrinsic resistance to IGF-1R targeted therapy.. Proc Natl Acad Sci U S A.

[16] Gao J, Chesebrough JW, Cartlidge SA, Ricketts SA, Incognito L (2011). Dual IGF-I/II-neutralizing antibody MEDI-573 potently inhibits IGF signaling and tumor growth.. Cancer Res.

[17] Weigelt B, Mackay A, A'hern R, Natrajan R, Tan DS (2010). Breast cancer molecular profiling with single sample predictors: A retrospective analysis.. Lancet Oncol.

[18] Paik S, Shak S, Tang G, Kim C, Baker J (2004). A multigene assay to predict recurrence of tamoxifen-treated, node-negative breast cancer.. N Engl J Med.

[19] Perou CM, Sorlie T, Eisen MB, van de Rijn M, Jeffrey SS (2000). Molecular portraits of human breast tumours.. Nature.

[20] Sotiriou C, Pusztai L (2009). Gene-expression signatures in breast cancer.. N Engl J Med.

[21] Marcom PK, Isaacs C, Harris L, Wong ZW, Kommarreddy A (2007). The combination of letrozole and trastuzumab as first or second-line biological therapy produces durable responses in a subset of HER2 positive and ER positive advanced breast cancers.. Breast Cancer Res Treat.

[22] Creighton CJ, Casa A, Lazard Z, Huang S, Tsimelzon A (2008). Insulin-like growth factor-I activates gene transcription programs strongly associated with poor breast cancer prognosis.. J Clin Oncol.

[23] Belfiore A, Pandini G, Vella V, Squatrito S, Vigneri R (1999). Insulin/IGF-I hybrid receptors play a major role in IGF-I signaling in thyroid cancer.. Biochimie.

[24] Jones HE, Gee JM, Barrow D, Tonge D, Holloway B (2006). Inhibition of insulin receptor isoform-A signalling restores sensitivity to gefitinib in previously de novo resistant colon cancer cells.. Br J Cancer.

[25] van 't Veer LJ, Dai H, van de Vijver MJ, He YD, Hart AA (2002). Gene expression profiling predicts clinical outcome of breast cancer.. Nature.

[26] Parker JS, Mullins M, Cheang MC, Leung S, Voduc D (2009). Supervised risk predictor of breast cancer based on intrinsic subtypes.. J Clin Oncol.

[27] Fan C, Oh DS, Wessels L, Weigelt B, Nuyten DS (2006). Concordance among gene-expression-based predictors for breast cancer.. N Engl J Med.

[28] Oh DS, Troester MA, Usary J, Hu Z, He X (2006). Estrogen-regulated genes predict survival in hormone receptor-positive breast cancers.. J Clin Oncol.

[29] Cheang MC, Chia SK, Voduc D, Gao D, Leung S (2009). Ki67 index, HER2 status, and prognosis of patients with luminal B breast cancer.. J Natl Cancer Inst.

[30] Papa V, Pezzino V, Costantino A, Belfiore A, Giuffrida D (1990). Elevated insulin receptor content in human breast cancer.. J Clin Invest.

[31] Milazzo G, Giorgino F, Damante G, Sung C, Stampfer MR (1992). Insulin receptor expression and function in human breast cancer cell lines.. Cancer Res.

[32] Vella V, Pandini G, Sciacca L, Mineo R, Vigneri R (2002). A novel autocrine loop involving IGF-II and the insulin receptor isoform-A stimulates growth of thyroid cancer.. J Clin Endocrin Metab.

[33] Malaguarnera R, Frasca F, Garozzo A, Giani F, Pandini G (2001). Insulin receptor isoforms and insulin-like growth factor receptor in human follicular cell precursors from papillary thyroid cancer and normal thyroid.. J Clin Endocrin Metab.

