# Supplementary material for: Altered Expression of Insulin Receptor Isoforms in Breast Cancer
Source: PLoS One. 2011 Oct 26;6(10):e26177. doi: 10.1371/journal.pone.0026177 (PMC3202518; doi:10.1371/journal.pone.0026177)
Supplement: Table S1 — Specificity of IR-A and IR-B assays. Assay specificity was tested by performing IR-A, IR-B, and IR qRT-PCR assays with a serial dilution of approximately 107 to 10 copies of plasmid DNA of IR-A and IR-B. Cross reactions of either IR-A assay to IR-B DNA or IR-B assay to IR-A DNA were not observed. (PDF) [file pone.0026177.s001.pdf]

Supplementary Table 1. Specificity of IR-A and IR-B assays

IR-A Specificity

IR-A		AVG C_T			STDEV		
Total DNA Amount (pg)	DNA Copy Number	IR_{assay}	$IR\text{-}A_{assay}$	$IR\text{-}B_{assay}$	IR_{assay}	$IR\text{-}A_{assay}$	$IR\text{-}B_{assay}$
100	9200000	16.96	15.61	ND	0.18	0.09	NA
50	4600000	17.91	16.26	ND	0.05	0.05	NA
25	2300000	18.25	16.78	ND	0.00	0.00	NA
12.5	1150000	19.21	17.70	ND	0.20	0.00	NA
6.25	575000	20.58	18.88	ND	0.02	0.01	NA
3.13	287500	21.57	19.84	ND	0.08	0.07	NA
0.78	71875	23.40	21.58	ND	0.02	0.02	NA
0.20	17969	25.41	23.70	ND	0.04	0.08	NA
0.10	8984	26.49	24.82	ND	0.01	0.07	NA
0.05	4492	27.42	25.69	ND	0.04	0.10	NA
0.012	1123	29.66	27.85	ND	0.14	0.05	NA
0.006	562	30.47	28.82	ND	0.32	0.05	NA
0.0015	140	32.45	30.61	ND	0.15	0.16	NA
0.0004	35	34.42	32.48	ND	0.27	0.26	NA
0.0001	9	ND	ND	ND	NA	NA	NA

IR-B Specificity

IR-B		AVG C_T			STDEV		
Total DNA Amount (pg)	DNA Copy Number	IR_{assay}	$IR\text{-}A_{assay}$	$IR\text{-}B_{assay}$	IR_{assay}	$IR\text{-}A_{assay}$	$IR\text{-}B_{assay}$
100	9540000	15.20	ND	14.54	0.05	NA	0.06
50	4770000	15.87	ND	14.98	0.01	NA	0.04
25	2385000	16.53	ND	15.17	0.02	NA	0.01
12.5	1192500	17.27	ND	16.04	0.01	NA	0.06
6.25	596250	18.23	ND	16.85	0.11	NA	0.03
3.13	298125	19.09	ND	17.65	0.05	NA	0.05
0.78	74531	20.80	ND	19.63	0.03	NA	0.03
0.20	18633	23.10	ND	21.56	0.09	NA	0.01
0.10	9316	23.97	ND	22.32	0.03	NA	0.01
0.05	4658	24.77	ND	23.25	0.18	NA	0.06
0.012	1165	27.15	ND	25.59	0.06	NA	0.05
0.006	582	28.03	ND	26.50	0.12	NA	0.33
0.0015	146	30.42	ND	28.29	0.16	NA	0.08
0.0004	36	32.14	ND	30.19	0.12	NA	0.08
0.0001	9	ND	ND	ND	NA	NA	NA

ND = not detected; NA = not applicable
